# Resilient Multiuser Session Control in Softwarized Fog-Supported Internet of Moving Thing Systems

**DOI:** 10.3390/s19122766

**Published:** 2019-06-20

**Authors:** Helber Wagner da Silva, Augusto José Venâncio Neto

**Affiliations:** 1 Department of Informatics, Federal Institute of Education, Science and Technology of Rio Grande do Norte (IFRN), Canguaretama 59.190-000, Brazil; 2 Department of Informatics and Applied Mathematics (DIMAp), Federal University of Rio Grande do Norte (UFRN), Natal 59.078-970, Brazil; augusto@dimap.ufrn.br

**Keywords:** Internet of Moving Things, mission-critical, IoT applications, resilient quality-oriented multiuser control, softwarized network control plane

## Abstract

The combination of IoT and mobility promises to open a new frontier of innovations in smart environments, through the advent of the Internet of Moving Things (IoMT) paradigm. In IoMT, an array of IoT devices leverage IP-based mobile connectivity to provide a vast range of data ubiquitously. The IoMT realization will foster smart environments at unprecedented levels, by efficiently affording services and applications whereby today’s technologies make their efficiency unfeasible, such as autonomous driving and in-ambulance remotely-assisted patient. IoMT-supported mission-critical applications push computing and networking requirements to totally new levels that must be met, raising the need for refined approaches that advance beyond existing technologies. In light of this, this paper proposes the Resilient MultiUser Session Control (ReMUSiC) framework, which deploys emerging softwarization and cloudification technologies to afford flexible, optimized and self-organized control plane perspectives. ReMUSiC extends our previous work through the following innovations. A quality-oriented resilience mechanism is capable of responding to network dynamics events (failure and mobility) by readapting IoMT multiuser mobile sessions. A softwarized networking control plane that allows to, at runtime, both fetch current network state and set up resources in the attempt to always keep affected IoMT multiuser mobile sessions best-connected and best-served. A cloudification approach allows a robust environment, through which cloud- and fog-systems interwork to cater to performance-enhanced capabilities. The IoMT’s suitability and performance impacts by ReMUSiC framework use are assessed through real testbed prototyping. Impact analysis in Quality of Service (QoS) performance and perceived Quality of Experience (QoE), demonstrate the remarkable abilities of the ReMUSiC framework, over a related approach, in keeping IoMT multiuser mobile sessions always best-connected and best-served.

## 1. Introduction

The technological evolution of the Internet of Moving Things (IoMT), which advents from the combined assistance between IoT and network mobility, promises to influence people’s daily lives (regardless of whether they are aware or not) by providing quick and useful information about the environment and the people themselves. The IoMT paradigm denotes an array of IoT devices that leverage IP-based mobile connectivity to provide a vast range of data ubiquitously. In the next decade, the realization of the IoMT is foreseen to be able to influence how people dress or behave, how industries increase their productivity, and how cities offer public services to citizens [1]. To that end, IoMT is based on the connectivity between people, systems and a lot of moving devices (e.g., sensors, actuators, vehicles, and many other potentially moving things) anywhere, anytime, from applications that fit the user needs. On the one hand, the success of IoMT will occur due to the growth in quantity (it is expected 12.3 billion in 2022 [2] of moving things silently integrated into people’s daily lives. On the other hand, the numerous and emerging IoMT applications (i.e., applications supported by the resources available in IoMT systems) have been developed in a wide range of areas, including transport, healthcare, safety, environmental preservation, with the potential to benefit people in many dimensions, including reduced living costs, improved personal comfort, sustainable and ecological development and greater physical security.

Among the many emerging IoMT applications, the most significant advances in the next few years should occur in the so-called Critical Mission IoMT Applications (CMIA), which promise impacts in many vital areas. Some of the promising CMIAs in the coming years include the following:

- Smart surveillance. These CMIAs are classified as of “very hot” interest by favouring very rapid interventions through public devices (e.g., police, fire, ambulance etc.) in the face of potentially critical circumstances (e.g., natural catastrophes, vehicle accidents, terrorism etc.) in a densely populated environment [3];
- Smart transportation. A smart transport CMIA with a high impact, especially for the general population involves road safety. The CMIA should be developed to ensure the detection and removal of objects, animals or people on densely used roads, particularly high-speed roads, in the shortest possible time, to ensure the safety of users while they travel with their vehicles [4]; and
- Tele-surgery. Remote surgery CMIAs allow, in emergencies where time wastage is a determining factor in increasing a patient’s life risk, a less experienced surgeon on the side of the patient can be assisted by video by a specialist away from the surgery environment [5].

Although CMIAs are beneficial in different areas, some characteristics are familiar to many of them. Firstly, CMIAs execute their decision-making processes through typically complex and costly algorithms that need a very short time and enhanced accuracy [6]. Secondly, the decision-making approach of multimedia-based CMIAs (as listed hereinabove) need to process received content (video and/or audio) in (near to) real-time [7]. Lastly, CMIAs decision-making approach can be classified as time-critical, especially in emergencies (e.g., the threat of death or serious physical harm), whereby procedures need to be enforced immediately on detecting. These fundamental characteristics differentiate CMIAs themselves from other IoMT applications.

CMIAs have a high demand for network resources (e.g., processing/storing network elements or nodes, paths, bandwidth etc.) and are highly sensitive to the variation of Quality of Service (QoS) and Quality of Experience (QoE) [8]. The CMIA-relevant key QoS performance indicators include latency, jitter, throughput, and packet loss rates. Video-based streaming CMIAs, for example, require maximum latency of 5 s and loss rates of 5%, which need to be ensured by the network [9]. Other interactive video-based CMIAs have even more stringent QoS requirements, which include maximum latency (150 ms), maximum jitter (30 ms), and loss rate (1%) [10]. However, the importance of each of these QoS requirements may vary, depending on the nature of the CMIAs. For example, in Smart Surveillance, the quality of decisions (e.g., triggering public safety services or braking a stand-alone car in the face of an obstacle on the road) can be strongly impacted by reducing the flow of content delivery. For other highly interactive CMIAs, such as tele-surgery, latency is one of the most relevant QoS requirements. Thus, the QoS aspects of being ensured by the network depend on the QoS requirements associated with the content required by the CMIA [11].

However, in the current communication session model between CMIA/IoT platform, each CMIA content request will be handled (by most) cloud-running IoT platforms, individually [12]. This leads to an inefficient content transport service in IoMT due to different reasons: (i) it wastes computational resources (e.g., processing and storage) on the cloud IoT platform; (ii) it induces a potentially exponential amount of redundant micro-flows (i.e., individual one-to-one flows) that drain network resources and increase the packet loss rate in the links; (iii) it limits scalability, by reducing performance when multiple CMIAs request content with the same characteristics at a time; and (iv) it leads to a breach of the quality requirements of CMIAs.

Some recent solutions have attempted to ensure QoS at the network level, including group-based communication strategies. Classic solutions have been regularly based on IP Multicast group communication service over SDN network (denoted SDN Multicast in this paper), in which selected SDN nodes are enforced with group-based forwarding rules. Although these solutions can optimize data communication within an SDN domain, they are limited in the IoMT. Firstly, they reduce the degree of interoperability and increase the complexity of CMIAs, which need to know a priori (i.e., before submitting a content request to the cloud IoT platform) details of the network level (i.e., IP Multicast address to join). Secondly, QoS-oriented content transport based on SDN Multicast solutions focuses only on network layer parameters (being completely unaware of the content required by the CMIAs) to build the network paths, which makes it impossible to refine resource allocation services (e.g., paths, bandwidth etc.) and more optimized, considering the knowledge of the required content [13].

Among the wide multidimensional system innovation range that the 5G network advent promises, the Ultra Reliable and Low Latency Communications (URLLC) futuristic use case attains special attention in the CMIA scope, by the pursuit incredibly latency rates of less than 1 ms [14]. Indeed, the alliance between cloud computing and IoT (a.k.a. IoT cloudification) is tremendously beneficial for CMIAs, through provisioning remote computing and storage powers, security and privacy, interoperability and others services in an on-demand delivery approach. However, cloud-supported IoMT poses issues in many different fields, as in the following. Firstly, enormous network costs arise with things regularly posting sense data to remote Cloud infrastructure. The cloud computing centralized approach stands inappropriate to the timely processing of large data volumes that CMIA holds.

Moreover, the scalability of cloud infrastructures exponentially degrades with the massive-density of moving things, impacting the ability to keep the required CMIA QoS [15]. Leveraging computing capabilities at network edges to enable gathered data to be either on-site processed or providing any timely assistance instead of sending down to a remote cloud infra, arises as a prominent solution to cater time-critical decision makings [16]. Fog computing is founded on the premise of extending cloud computing capabilities to the network edges, in a sense to enable the computing-distributed approach. In other words, network edge nodes deploy additional layers devoted to on-site data processing. In addition to remote data storage, fog nodes interwork with the cloud to leverage further refined both processing and analytics services.

Based on all the IoMT concepts mentioned above, in terms of both benefits and challenges, the main problem addressed in this paper is how to efficiently offer complex and highly dynamic CMIAs holistically. Our investigation seeks answers to the following research questions: (i) how to handle network dynamics that affect the CMIA quality in the IoMT in a timely critical way?; and (ii) is it possible to refine the integration between CMIAs and cloud computing catering performance-enhanced perspectives? Considering the problem to be solved and the issues raised, this work is based on the following hypothesis: it is possible to provide quality-oriented resilient content transport considering a set of IoMT multiuser mobile sessions in which different fog nodes connect over a holistic, softwarized and cloudified control plane that advances beyond current IoT technology capabilities. In light of this, we propose the Resilient MultiUser Session Control (ReMUSiC) framework, which is based on the softwarized substrate to orchestrate, at runtime, fog-connecting QoS-guaranteed IoMT multiuser mobile sessions in a self-organized way. ReMUSiC features a modular architecture, whereby cloud- and fog-supported participating components interwork to provision IoMT multiuser mobile sessions, and enforce procedures at runtime to timely respond to network dynamics, seeking to keep affected IoMT multiuser mobile sessions always best-connected and best-served. The IoMT’s suitability and performance impacts by ReMUSiC framework use are assessed through real testbed prototyping. Impact analysis in QoS performance and perceived Quality of Experience (QoE), demonstrate the remarkable abilities of the ReMUSiC framework, over a related approach, in keeping IoMT multiuser mobile sessions always best-connected and best-served.

### Outline

The remainder of this paper is structured as follows. Section 2 discusses the recent research that seeks to optimize the content transport in IoMT, identifying its main limitations. Section 3 describes the ReMUSiC framework. Section 4 presents the results obtained by performing experiments on a real testbed, comparing resilience and performance of ReMUSiC to a solution based on SDN Multicast in a video scenario. In Section 5 we conclude the paper and suggest future investigations.

## 2. Related Works

Several studies have attempted to propose solutions to provide QoS-guaranteed data transmission at IoT over Software Defined Networks (SDN), thus including SDN-enabled QoS solutions [17]. The most commonly used communication models are unicast, multicast and group-based. Unlike IP multicast-based solutions, SDN group-based communication relies on SDN substrate to replicate packets in the direction of multiple destination (e.g., where CMIAs are executed) host(s) of a data stream.

The work of Llopis et al. [18] proposes a unicast-based solution to select SDN paths with lower latency for IoT applications. This solution implements a candidate path discovery mechanism, a latency measurement system, and a path selection algorithm. The path discovery mechanism runs on the SDN Controller to identify candidate paths. The latency measurement system periodically induces probe packages to measure the value of Round Trip Time (RTT) of each candidate path. After these measurements, an algorithm selects the path with the lowest latency for the data traffic.

Chen et al. [19] propose a routing algorithm based on the multipath approach to ensure QoS requirements (latency) specified by IoT applications. The algorithm seeks to optimize the power consumption of the nodes and to reduce the interference between paths in the multimedia transmission between sensors and the sink node in Wireless Sensor Networks (WSN). The algorithm employs a mathematical model that estimates the number of hops to guarantee the transmission latency requirement, considering the access control scheme to the Distributed Contention Function (DCF) of the IEEE 802.11b standard.

Recently, other solutions have been based on IP multicast to provide group communication over the SDN network. Huang et al. [20] proposed a routing solution based on SDN/OpenFlow Multicast to satisfy QoS requirements, specifically packet loss rate and latency in the transmission of traffic flows. It calculates multicast trees formed by paths with greater residual bandwidth. The bandwidth of the links is monitored by a module that uses the sFlow monitoring tool in each OpenFlow switch. This information is then forwarded to the SDN Controller (Ryu), which interacts with the routing solution to select the best paths in terms of residual bandwidth associated with the multicast groups.

The OpenE2EQoS algorithm [21] seeks to perform a bandwidth provisioning to ensure QoS data transmission. It (re)dynamically allocates bandwidth to accommodate multiple hosts associated with multimedia multicast groups (i.e., whose traffic loads multimedia data). The algorithm uses a predictive model to meet the multimedia multicast group’s QoS requirements. When a link in a multicast tree path associated with a group exhibits congestion, the algorithm statistically distributes packets with low priority between a set of alternative and lower quality paths. Soni et al. [22] also proposed a multicast SDN-based service for multimedia traffic, whose main objective is to provide QoS (bandwidth) and scalability in the softwarized networks of Internet Service Providers. This service seeks to avoid the exponential growth of the number of messages in the IGMP protocol due to the increase in the scale of the ISP’s network, by using a virtual network function that delegates the management of the multicast groups for nodes closest to the edge of the network.

These solutions are limited in the IoMT scenarios due to the decoupled view between cloud IoT platforms and CMIAs. This decoupling is revealed by the following inherent characteristics of the IoMT. Firstly, CMIAs use top-level protocols when requiring content from cloud IoT platforms [23], while SDN Multicast solutions require an application to explicitly request its interest in joining a previously known multicast group. Secondly, cloud IoT platforms do not expose services to refine the content transport in softwarized networks [12]. This makes cloud IoT platforms incapable of inducing quality-guaranteed content transport towards multiple CMIAs.

The works in [21,22] use mechanisms to monitor network dynamics, allowing them to react in the adaptation of the SDN Control Plane in a resilient way. However, none of these works (other than ReMUSiC) implements a holistic, resilient IoMT multiuser mobile session view capable of providing resilient content transport with guaranteed quality in IoMT scenarios, even in the face of network dynamics.

Other solutions have been proposed using the group-based communication model on the SDN network, where one-to-many rules are installed on SDN nodes to replicate packets in the direction of multiple IoT applications. The SDM^2 Cast [24] framework, for example, constructs a multicast tree and an associated stream address for each video layer, adapting the trees to one-to-one entries in OpenFlow. In this framework, each node modifies the flow address with the IP address of the destination in the packet header, making it unnecessary to use the IGMP protocol. Based on link and port utilization statistics for SDN nodes, the framework can increase or reduce the number of video layers to fetch better QoE for end users.

In the work of Hungyo and Pandey [25], the authors proposed an OpenFlow-based solution to provide network support for the publish/subscribe model in SDN Multicast networks. This solution allocates clusters that group multiple subscribers associated with the same multicast group. The purpose of clustering is to reduce the latency in the dissemination of data to clusters and to optimize the occupation of flow tables in nodes.

The related work analysis exhibits that solutions can be categorized as based on unicast [18,19,26,27,28], in SDN Multicast [20,21,22,29,30,31,32] or in groups on SDN [24,25], according to the communication model used in the SDN data plane. The Table 1 summarizes the key features of these solutions in line with requirements, including: (i) session-level semantics, (ii) multiuser communication, (iii) quality-oriented control plan, and (iv) resilience.

It is possible to observe that most of the recent solutions are based on SDN Multicast, which, unlike solutions based on unicast, seek to eliminate packet redundancies in the network. This benefit can also be achieved by solutions based on the SDN group model, which has yet to be explored. For solutions based on SDN Multicast, they are limited in terms of: (i) inefficiency and low scalability due to multicast group management protocols; (ii) high complexity/overhead of signalling in the maintenance of multicast distribution trees by the network nodes; (iii) inefficient support to multicast by the hardware of the nodes; (iv) CMIAs need to know a priori network-level information, such as the multicast IP address, so that its host can do *join* in a multicast group; (v) inflexibility for dynamic adaptation of distribution trees to changes in the network (e.g., sensor mobility, link/node failure, traffic pattern etc.), among others [33].

It can be seen that the work of Noghani and Sunay [31], OpenE2EQoS [21] and Soni et al. [22] use network dynamics monitoring mechanisms, allowing to react in the adaptation of the SDN Data Plan in a resilient way. The SDM^2 Cast [24] framework and the work of Hungyo and Pandey [25] have similarity to the ReMUSiC framework proposed in this paper, by using SDN substrate to implement group-based communication. However, none of these works (other than ReMUSiC) implements a holistic solution with a resilient multiuser session view that can provide the transport of content with guaranteed quality and scalability, even in the face of network dynamics and mobility.

## 3. The ReMUSiC Framework Proposal

**Figure 1:**
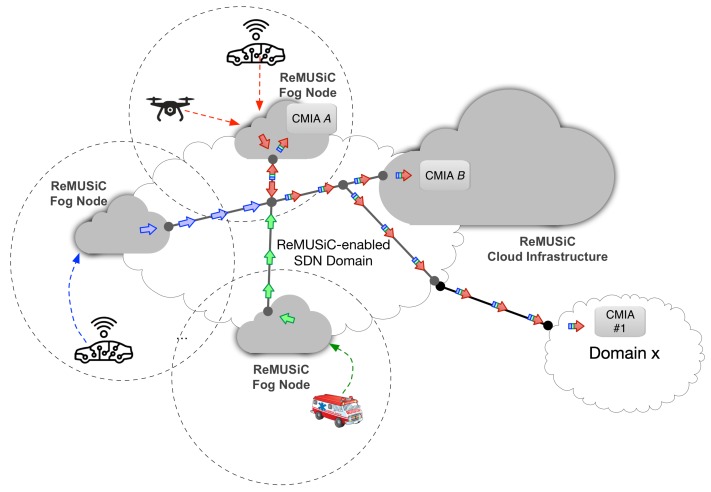
High-level of the Resilient MultiUser Session Control (ReMUSiC)-enabled ecosystem.

The ReMUSiC framework performs from our previous work [34], significantly extending the Cross-LAyer Sdn SessIon COntrol (CLASSICO) approach through innovations that entail new capabilities tailored for quality-oriented and resilient multiuser communication resource control inside IoMT-supported domains. On the one hand, CLASSICO solution provides the baseline approach to build multiuser communication sessions along with edge-to-edge OpenFlow-enabled links inside the respective domain. The ReMUSiC, on the other hand, significantly advances on the basis of providing control plane innovations tailored to dynamically provisioning IoMT multiuser mobile sessions through the following functions: (i) a quality-oriented resilience mechanism capable of responding to network dynamics events (failure and mobility) by readapting IoMT multiuser mobile sessions; (ii) a softwarized networking control, that allows to both fetch current network state and setup resources, at runtime, in the attempt to keep affected IoMT multiuser mobile sessions always best-connected and best-served; (iii) a cloudification infrastructure, through which cloud- and fog-systems interwork to cater performance-enhanced capabilities to support IoMT multiuser mobile sessions. Figure 1 presents a high-level of the ReMUSiC-enabled ecosystem.

**Figure 2:**
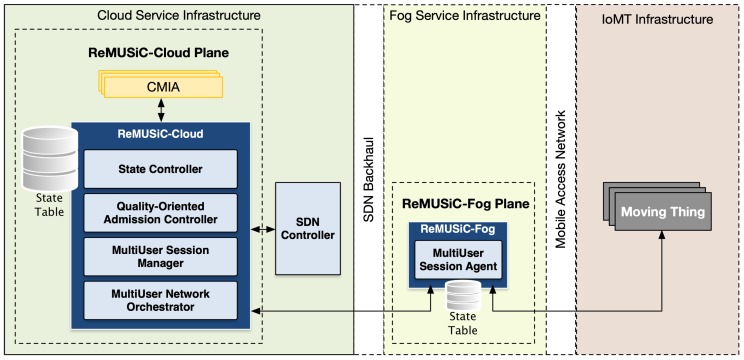
ReMUSiC’s internal architecture in the Internet of Moving Things (IoMT) scenario.

The ReMUSiC framework follows a holistic approach that integrates a resilient, self-organized and flexible softwarized control plane to afford quality-guaranteed content transport provisioning for Critical Mission IoT Applications (CMIAs) in IoMT scenarios. Following essential capability, sets integrate the service provisioning approach of the ReMUSiC framework: (i) quality-oriented admission control; (ii) self-organized quality-oriented management of IoMT multiuser mobile sessions leveraging network-softwarized substrate; and (iii) quality-oriented resilient mobile content transport. Such services interoperate with CMIAs and fog nodes transparently towards ensuring flexibility. The ReMUSiC framework deploys a functional architecture that follows a modular approach, whereby each participating component interoperates with every other and with other entities using internal and external interfaces, respectively. Figure 2 presents a high-level view of the ReMUSiC functional architecture, along with non-exhaustive control plane interactions among internal participating components and external enabling technologies.

Based on the functional architecture that Figure 2 depicts, the ReMUSiC’s control plane is split into two fundamental systems, according to respective locations positioned in the IoMT scenario:

- ReMUSiC-Cloud: running in the cloud service infrastructure inside the ReMUSiC-enabled ecosystem, the following components participate: State Controller, Quality-oriented Admission Controller, MultiUser Session Manager and MultiUser Network Orchestrator. A state table is set to provide all necessary knowledge to the ReMUSiC-Cloud plane;
- ReMUSiC-Fog: running in every available fog system composing the ReMUSiC-enabled ecosystem, counts with the participation of the MultiUser Session Agent, assisted by state table to provide local-premise knowledge.

In regards to the assumptions behind this work, ReMUSiC-Cloud relies on existing resource discovery services on cloud IoT platforms [35] to share knowledge about available IoMT devices along with attached fog nodes inside the ReMUSiC-enabled domain. Moreover, the announcement of activated IoMT publishing content knowledge in the pursuit to receive interested CMIA subscribers, as well as the service orchestration procedures in both cloud and fog systems, are tasks assumed to be on the service operator. Finally, all the hereinabove assumptions and the guidelines to choose the best enabling technologies and tools is totally out of the scope of this paper.

In order to contextualize the functional architecture along with all participating components, a basic workflow exemplifies the ReMUSiC framework interworking operations as in the following. On receiving a content request message from a CMIA exposing intent to receive content that a session activated in a fog node publishes, the ReMUSiC-Cloud subsystem carries out the set of operations as below in a non-exhaustive way:

1. the quality-oriented admission control service decides to accept (or not) the session request, taking as input the session requirements along with current QoS network conditions to access the respective fog node;
2. on admitting, an IoMT multiuser mobile session is created (or adapted) to add the intended CMIA as an IoMT multiuser mobile session participant;
3. afterwards, the best network path is selected (with the assistance of the topology management service provided by the SDN Controller) to connect the fog node (content provider) and all the CMIA participants in the IoMT multiuser mobile session, as well as the SDN node nearest to the corresponding fog node, which will replicate each packet associated with the content in the direction to all CMIAs.

All components use a representation of the *internal state* of the resilient and quality-oriented content transport service. The internal state corresponds to the updated information about the resources available at the IoMT multiuser mobile session and the network levels, which can be managed (e.g., allocated or reallocated) dynamically for multiple CMIAs simultaneously. In this work, such resources include: (i) at the IoMT multiuser mobile session level, the session participants and content associated with an established session; (ii) bandwidth on physical links, and network paths connecting fog systems to each output node (called *egress node*) of the softwarized network on the way to a CMIA.

Semantic knowledge of an IoMT multiuser mobile session, along with an illustrative example, is shown in Table 2 whereby an IoMT multiuser mobile session is structured in the form of a tuple with the following fields: *(Identifier, Producer, Content, Participants)*.

Each tuple field has a corresponding semantics that identifies an IoMT multiuser mobile session. The semantics of each field is described as follows. The ReMUSiC-Cloud system sets, to each activating IoMT multiuser mobile session, an unambiguous exclusive *Identifier*, being represented by a value of integer type. The *Producer* corresponds to an IoMT application, running in the form of virtualized structure (e.g., container) of a given fog node inside the ReMUSiC-enabled domain, that publishes corresponding content (that can be either raw data produced by attached IoMT devices or information analytics). The *Content* field is filled with session content specifications, which is assumed to be accessible through the assistance of the cloud IoT platform features. Such content specifications include the following parameters: name, type, value, video codec specifications (e.g., H.264, Motion JPEG, Theora, VP8 etc.), audio codec (e.g., Advanced Audio Coding, Vorbis, etc.), video format, bit-rate, among others. ReMUSiC can obtain these properties through a content descriptor API of the IoT [36] or through the exchange of negotiation messages (e.g., Session Session Protocol (SDP) protocol [37]) with the IoT platform. The *Participants* are the set of all the participants of the IoMT multiuser mobile session, each one being represented by the pair IP address + port of the transport level associated with the CMIA.

In the example of Table 2, there are three different IoMT multiuser mobile sessions in the internal state, whose identifiers (*Identifier*) are equal to 1, 2 and 3. Each IoMT multiuser mobile session allows sharing a *Content* described as $video_{1}$, $video_{2}$ and $video_{3}$, respectively, whose processed information are provided by each fog system *Producer* with the addresses $id_{fog_{1}}$, $id_{fog_{2}}$ and $id_{fog_{3}}$. The *Participants* of each IoMT multiuser mobile session are described as follows: $\left\{cmia_{1},cmia_{2}\right\}$ are with IoMT multiuser mobile session 1, $\left\{cmia_{3},cmia_{4},cmia_{5}\right\}$ are with IoMT multiuser mobile session 2 and $\left\{cmia_{6},cmia_{7}\right\}$ are associated to IoMT multiuser mobile session 3.

### 3.1. ReMUSic-Cloud Components and Modules

This section details all components and modules participating in the ReMUSiC-Cloud’s internal architecture, highlighting objectives, procedures, structures, and interactions with other entities in IoMT.

#### 3.1.1. State Controller

**Figure 3:**
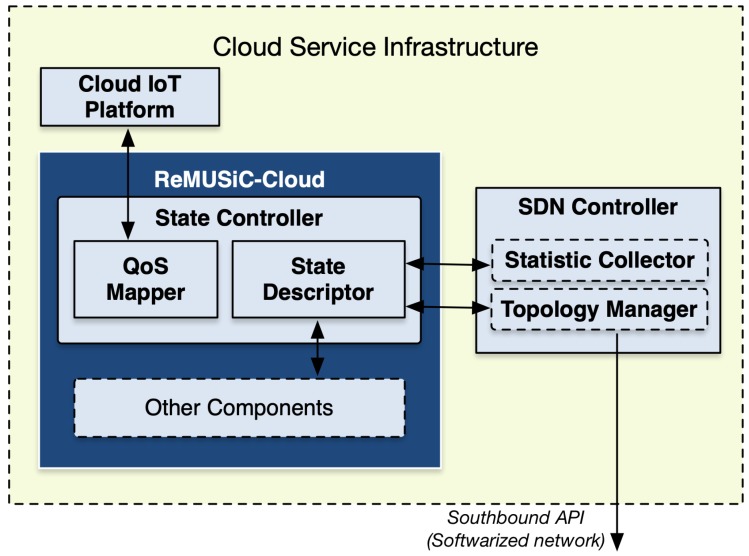
Internal architecture of the State Controller (SC) component.

The State Controller (SC) component is responsible for managing the internal state. Its main tasks include to: (i) obtain the most up-to-date knowledge on available resources; and (ii) expose the internal state to the other components. For this, the SC executes procedures implemented by modules, shown in Figure 3.

As part of the SC component, the *State Descriptor* module is responsible for obtaining, updating, and making available the internal state, employing interactions with the SDN Controller and other components. The *QoS Mapper* module builds a *QoS knowledge* associated with a certain content exposed by the platforms in IoMT. Such knowledge corresponds to the mapping of content properties to a set of QoS requirements (e.g., minimum bandwidth, maximum latencies/jitter/loss etc.) at the network level, which needs to be ensured in transporting that content. The next subsections detail each module.

##### State Descriptor

As the content transport service is provided, the tendency is that the internal state needs to be updated in the face of variations in such values due to the following events: (i) creation or grouping of IoMT multiuser mobile sessions; (ii) selection of paths for the transport of content in the softwarized network; (iii) change in network topology (e.g., failure, inclusion, removal, activation and/or deactivation of fog nodes/moving things and/or links, among others); or (iv) change in network behavior (e.g., increase in traffic on the links that decreases residual bandwidth, retransmissions that increase latency in the paths etc.). These modifications are notified to the State Descriptor using triggers with other entities in IoMT. Since such values can be obtained directly in the IoMT Control Plane, there is no additional signalling traffic on the network. The information exchange takes place using interfaces that implement the following triggers:

1. The statistics collection service (provided by the SDN Controller) passes the new values of the statistics at the network level in the paths, in front of events that change these values;
2. The topology management service (also provided by the SDN Controller) passes the set of all available paths when a fault occurs (which in this paper corresponds to any event that causes a change in the network topology);
3. The MultiUser Session Manager component (described in Section 3.1.3) invokes the State Descriptor, which reflects in the internal state the creation or grouping of IoMT multiuser mobile sessions; and
4. The MultiUser Network Orchestrator component, which is described in Section 3.1.4, invokes the State Descriptor to update the internal state with the paths selected for content transport in the softwarized network.

When the State Descriptor notifies triggers 1 or 2 of the hereinabove list, it induces the execution of a resilience procedure [38], executed by the Path Selector component (described in Section 3.1.4). This procedure aims to implement the resilience service while seeking to maintain the content transport service with best-quality even before either the reduction of QoS at the network level or failures. In the case of triggers 3 and 4 of the list above, it directly updates the internal state.

##### QoS Mapper

The QoS Mapper module has the primary goal of building an anticipated QoS knowledge of content. This knowledge is obtained by mapping the properties of each content to their respective network-level QoS requirements. By anticipating this knowledge (i.e., obtaining it before a content requisition message sent from a CMIA reaches the cloud IoT platform), it is possible to quickly identify which requirements need to be met in transporting the content required by the CMIA. This avoids unnecessary delays (e.g., checking whether content exists in the cloud IoT platform, obtaining content information etc.) in carrying out the procedures.

After obtaining content information, a mapping function creates the set of network-level QoS requirements that need to be ensured. The idea behind this mapping is to characterize CMIA quality requirements in the form of QoS requirements, which depends on the behaviour of the flow of content in the network. For example, when content is associated with a real-time interactive video CMIA (e.g., remote surgery), the QoS Mapper converts to “Latency<150ms;Jitter<30ms”) format. The same reasoning applies to other content associated with multimedia conferencing, video, broadcast, etc. [39]. Content-aware QoS knowledge is made available to the Quality-oriented Admission Controller, detailed in the next subsection.

#### 3.1.2. Quality-Oriented Admission Controller

The Quality-oriented Admission Controller (QoAC) component implements the measurement- based quality-oriented admission control service. Its main objective is to decide whether or not to accept the quality-assured content transport service for a CMIA, centralizing the decision and considering network-level QoS (i.e., internal statistics) measurements and knowledge of the QoS associated with the required content. To do this, the QoAC interoperates with the SC’s modules.

The QoAC is activated on receiving a content request message, which is generated on the network when a CMIA invokes the delivery service exposed by an API on a cloud IoT platform. The content request message contains CMIA needs, which can be described using known formats (e.g., JSON, XML, HTML, among others) [40]. It is important to note that QoAC needs to intercept this message *before* it reaches the cloud IoT platform, allowing its decision-making procedures to be carried out. To do so, the SDN Controller must be instructed to pass all the content request messages to QoAC transparently, for both the CMIA and the cloud IoT platform, making QoAC interoperable in the IoMT.

In order to enable the transfer of content request messages to QoAC, the interactions between the Control Plan and the IoMT Data Plane are performed as follows. When the request is received by the ingress SDN node (*ingress node*) in the softwarized network, a flow rule lack event occurs (e.g., *table-miss* in OpenFlow). In this circumstance, the ingress node encapsulates the request in a signalling message (e.g., OpenFlow OFPT_PACKET_IN) and forwards it to the SDN Controller. Upon receipt of the signalling message, the SDN Controller inhibits standardized procedures (i.e., calculating the shortest path from the SDN joining node to the packet destination, installing one-to-one flow rules on nodes along the calculated path, path selection, etc.). These procedures will be executed later and in an optimized way by ReMUSiC-Cloud if the QoAC *accepts* the quality-assured content transport service. Then, the SDN Controller decapsulates the request and passes it to the QoAC through an internal interface.

**Figure 4:**
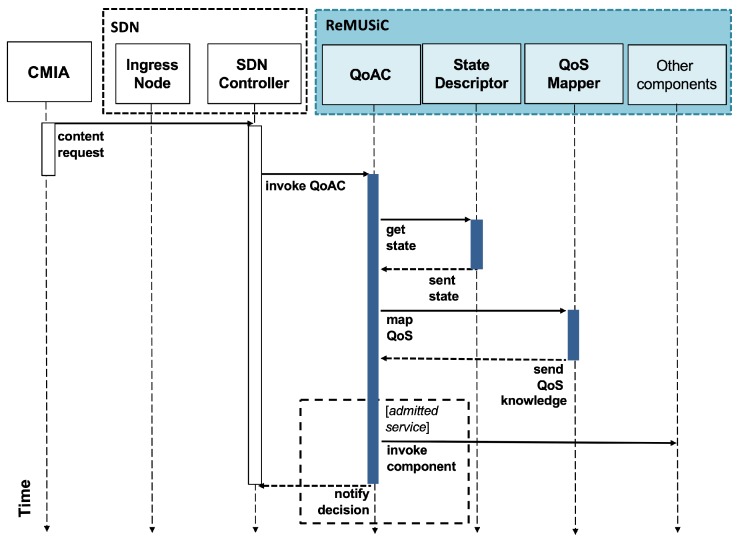
Sequence diagram of Quality-oriented Admission Controller (QoAC) procedures.

Figure 4 shows a sequence diagram of the QoAC procedures, which start when it receives the content request message from the CMIA (passed through the SDN Controller). This is the time when ReMUSiC-Cloud effectively starts provisioning the resilient content transport service with guaranteed quality. The QoAC procedures are implemented by the Algorithm 1.

**Algorithm 1:** Quality-oriented Admission Control.

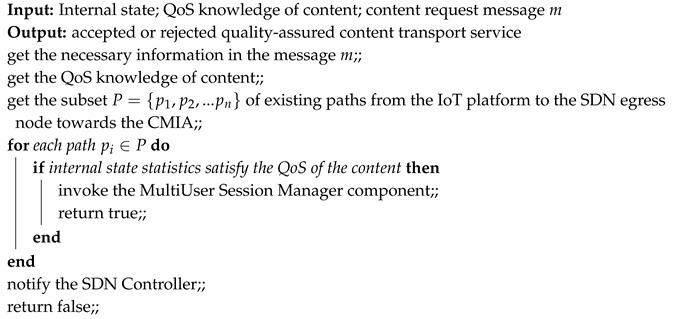



Algorithm 1 obtains the internal state and knowledge of the QoS associated with the content required by the CMIA, which make up its input. As a result, the algorithm invokes the ReMUSiC-Cloud MultiUser Session Manager component (described in Section 3.1.3, if the service is accepted, and returns the Boolean value *true*. Otherwise (i.e., if the service is rejected), the SDN Controller will receive the value *false*. The algorithm follows a cross-layer approach to decision making, by taking into account: (i) the description of the content required by the CMIA in the application-level headers of the request message; and (ii) the QoS statistics at the network level of the paths.

#### 3.1.3. MultiUser Session Manager

ReMUSiC-Cloud’s IoMT multiuser mobile sessions allow for a refinement of connectivity and interoperability control between multiple CMIAs and IoT platform, advancing against the limitations of the transport and network levels of the TCP/IP model. On the one hand, the level of TCP/IP transport basically builds transport units (segments) transported to the destination, using process identification ports at the application level. However, the TCP protocol flow control degrades network performance due to misinterpretation of congestion, especially when it is used in emerging and wireless networks [41]. On the other hand, the TCP/IP network level implements an unreliable service for transporting datagrams along the entire data path from source to destination (as well as IP Multicast, which uses protocol User Datagram Protocol for group content transport).

ReMUSiC induces optimized behaviour on the network, based on IoMT multiuser mobile session level knowledge. Instead of the cloud IoT platforms managing multiple requests of the same content (sent by different CMIA) independently, the Control Plan provided by ReMUSiC-Cloud allows it to manage only one request (i.e., the first CMIA that resulted in the creation of the IoMT multiuser mobile session), since subsequent requests from other CMIAs are inhibited by ReMUSiC through the management of active IoMT multiuser mobile sessions. In addition, knowledge of IoMT multiuser mobile sessions enables the optimization of network-based data transmissions based on group communication (from the cloud IoT platforms to the multiple CMIAs participating in each IoMT multiuser mobile session), which avoids redundant traffic and optimizes resource consumption from nodes/links.

In light of maintaining QoS for all accepted CMIAs, ReMUSiC-Cloud implements a self-organizing IoMT multiuser mobile session management service. This service is implemented by the MultiUser Session Manager (MUSM) component, which is responsible for adapting (i.e., allocating or relocating) the patterns for member composition of IoMT multiuser mobile sessions. MUSM can perform its procedures dynamically (i.e., at run time) and autonomously (i.e., without any human intervention), thus ensuring its self-organization.

MUSM initiates its procedures when invoked by QoAC. This occurs after the service admission to a CMIA when QoAC passes the content request message to the MUSM. It should be noted that until this moment of ReMUSiC-Cloud execution, this request message has not yet been passed through the SDN node in the IoMT Data Plane to the cloud IoT platform, thus supporting all other optimization services provided by the ReMUSiC framework.

**Figure 5:**
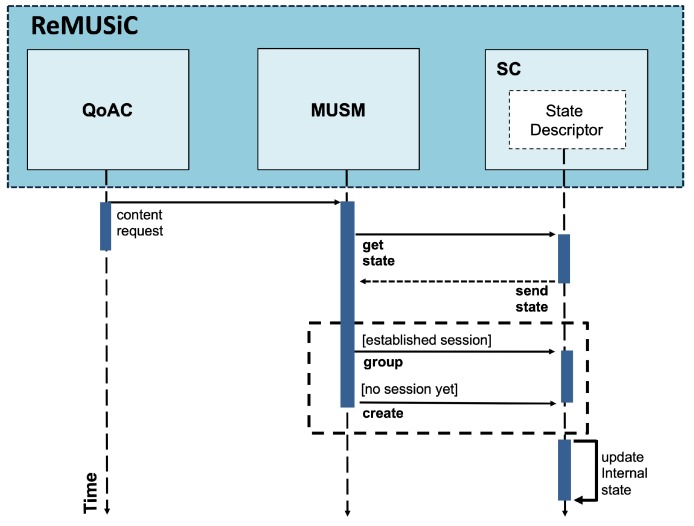
Sequence diagram of the procedures performed by the MultiUser Session Manager (MUSM) component.

Figure 5 shows a sequence diagram of the procedures performed by the MUSM component. When invoked (by QoAC), MUSM invokes the State Descriptor module of the SC component to obtain the internal state. Based on the required content (obtained in the application-level headers of the CMIA request message), MUSM checks for an active IoMT multiuser mobile session for the same intended content. If it exists, it induces clustering adaptation (i.e., the inclusion of the CMIA as a participant) in that IoMT multiuser mobile session. Otherwise, MUSM induces the creation of a new IoMT multiuser mobile session, including initially only the newly-accepted CMIA. After this procedure, MUSM invokes the State Descriptor module, informing: (i) the adapted session; and (ii) the IP address + transport-level port values associated with the CMIA, which then updates the internal state. In addition, the MultiUser Network Orchestrator (MUNO) component is notified to perform its specific procedures, based on the IoMT multiuser mobile session created or adapted.

#### 3.1.4. MultiUser Network Orchestrator

The SDN substrate exposed by the IoMT Control Plane offers the potential for multi-level optimization in the best support to CMIA [42]. ReMUSiC-Cloud benefits from this substrate, by orchestrating the selection of the best network paths for transporting the contents of IoMT multiuser mobile sessions. In this work, we consider that the best path is the one that provides the best balance between multiple QoS parameters (bandwidth, latency and jitter), which are appropriately kept updated in the internal state.

**Figure 6:**
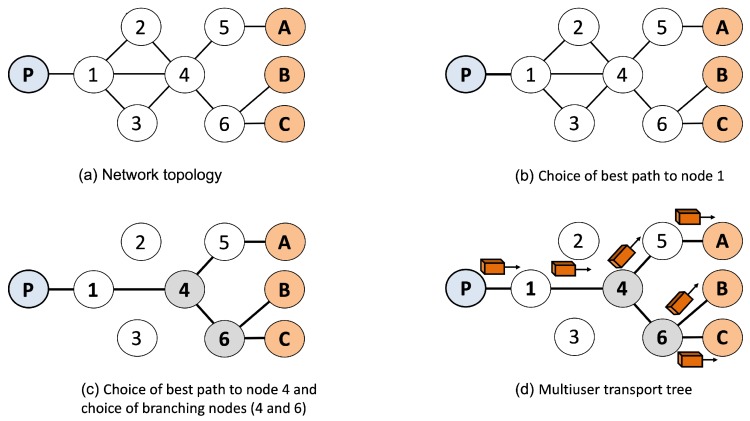
Multiuser transport tree.

The MultiUser Network Orchestrator (MUNO) component is responsible for mapping each IoMT multiuser mobile session in the form of a multiuser transport tree in the IoMT Data Plane. Each tree includes the best network paths, from the content provider (tree root) to each CMIA grouped in an IoMT multiuser mobile session. The set of all multiuser transport trees associated with all active IoMT multiuser mobile sessions form a multiuser softwarized network. Through these procedures, ReMUSiC-Cloud can implement a multi-level view (session and network) in IoMT in a holistic way. Figure 6 illustrates an example of a multiuser transport tree created by ReMUSiC-Cloud as a result of MUNO component procedures.

In the example, the network topology (letter (a) of Figure 6) connects the ReMUSiC-Fog node (content provider) *P* to the $cmia_{1}$, $cmia_{2}$ and $cmia_{3}$ consumers of the content. From this initial topology with multiple candidate paths from *P* to CMIAs, ReMUSiC-Cloud procedures build the multiuser transport tree that corresponds to an IoMT multiuser mobile session for the participants $\left\{cmia_{1},cmia_{2},cmia_{3}\right\}$. Initially, the MUNO component chooses the best path from *P* to the next hop (i.e., node 1) towards the CMIA (letter (b) in Figure 6). MUNO then chooses the best path to the node 4 and identifies that the nodes 4 and 6 can be chosen as branching nodes (letter (c)) in the multiuser tree to replicate data packets associated with the IoMT multiuser mobile session. Specifically, node 4 is branching for all three CMIAs, while node 6 is branching to both the $cmia_{2}$ and $cmia_{3}$. With the choice of branching nodes, the multiuser tree is built (letter (d) in Figure 6), and packet traffic carrying the shared content starts in the network.

The knowledge of multiuser transport trees (which is the basis for building the multiuser softwarized multiuser network) must be included in the internal state. To do this, it is necessary to combine: (i) the description of the multiuser transport trees; (ii) the semantic knowledge of the active IoMT multiuser mobile session of the internal state; and (iii) the statistics associated with QoS at the network level in the paths of the internal state. This combination of different knowledge allows ReMUSiC-Cloud to infer a new, more refined knowledge, i.e., the *quality-oriented IoMT multiuser mobile sessions*.

**Figure 7:**
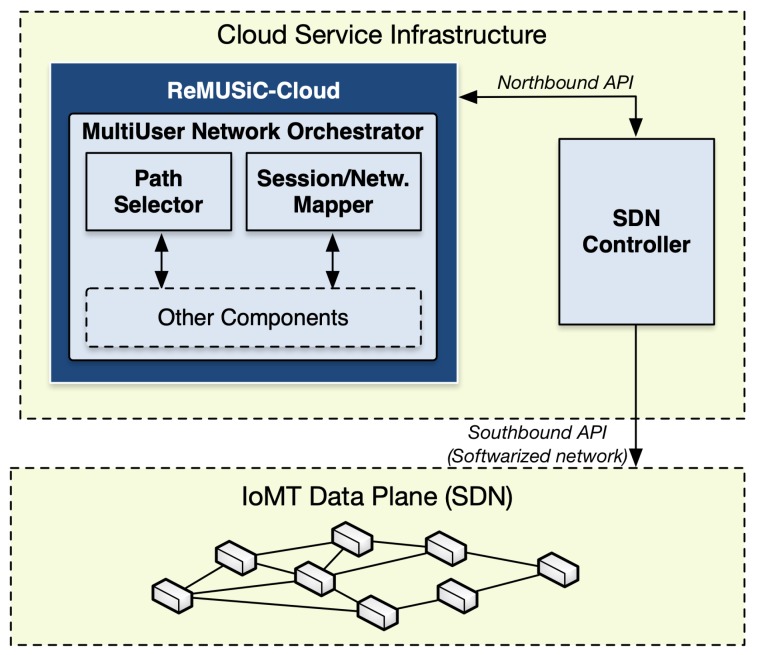
The MultiUser Network Orchestrator (MUNO) component.

Modules of their internal structure implement the procedures of the MUNO component, identified in Figure 7, whereby the MUNO component includes two modules: (i) *Path Selector* and (ii) *Session/Network Mapper*. These modules, respectively, (i) select the best paths to compose the multiuser transport trees, even in case network events or variations in QoS, and (ii) map the multiuser transport trees in the form of flow rules based on one-to-many groups (for branching nodes) and one-to-one for the other nodes. The following subsections detail the modules and their procedures.

##### Path Selector

The Path Selector module is primarily intended to select the best paths that define multiuser transport trees. To that, this module is invoked before the following events: (i) via the State Descriptor module, in case of network events or reduction of QoS in network paths, or (ii) via the MUSM component, at the end of the procedure of creating or adapting IoMT multiuser mobile sessions. In case of an event (i), the Path Selector performs a *path redundancy-based resiliency procedure*, which represents one of the network dynamics tolerance techniques [43], to maintain the quality-oriented content transport service. With the redundancy of paths, ReMUSiC-Cloud can dynamically select alternative paths (with guaranteed quality), if the path initially selected fails or reduces QoS.

The module uses a multi-criteria path selection metric, whose primary purpose is to balance multiple network-level QoS parameters, replacing the standard metric (number of hops) of the Dijkstra algorithm. This metric, named Performance Index (PI), allows classifying all candidate paths (according to the QoS they offer), which represent all those in the global topology between a content provider and the participants of an IoMT multiuser mobile session. The best paths are selected based on the PI value. The PI value of each path is calculated using a lightweight Multi-Criteria Decision Making (MCDM) method.

There are different MCDM methods that can be employed in decision processes. Examples of MCDM methods include [44,45,46,47,48]: Multi-Attribute Utility Theory (MAUT), Analytic Hierarchy Process (AHP), Fuzzy Set Theory, Case-based Reasoning, Data Envelopment Analysis, Simple Multi-Attribute Rating Technique, Goal Programming, ELECTRE, PROMETHEE e Technique for Order of Preference by Similarity to Ideal Solution (TOPSIS). Each MCDM method has advantages and disadvantages, and its use depends on factors, such as the type of application, the complexity involved in the decision-making process, the computational resources (e.g., processing and memory) available, among others. ReMUSiC-Cloud uses the Simple Additive Weighting (SAW) method to calculate the PI value of the paths, as the SAW method offers advantages over other existing MCDM methods, the ability to allocate weights that balance the importance of each evaluation criterion, avoiding inefficient decisions, and the use of simple mathematical operations (sums and multiplications), and therefore of low computational complexity.

In this work, the best path *p* for the quality-oriented content transport service is the one that offers the best balance (i.e., the one with the best PI value) considering available bandwidth ($awb_{p}$), latency ($delay_{p}$) and jitter ($jitter_{p}$). For each path p, the value of awbp corresponds to the lowest bandwidth available in the links, whereas the values $delay_{p}$ and $jitter_{p}$ represent the highest values of latency and jitter, respectively. These are the values that are stored in the internal state, as they limit QoS in the quality-oriented softwarized multiuser network.

The value of PI for a given path *p*, represented by $\varphi_{p}$, is defined by Equation 1. Based on the SAW method, the equation defines that the values $\bar{awb_{p}}$, $\bar{delay_{p}}$ and $\bar{jitter_{p}}$ correspond, respectively, to the normalized values of available bandwidth, latency, and jitter of path *p*. This normalization considers the lowest value of available bandwidth and the highest latency and jitter values of all topology paths, being implemented using low computational complexity [49]. In Equation (1), the higher the latency value and/or the jitter, the lower the path quality, which is why the inverse of $\bar{delay_{p}}$ and $\bar{jitter_{p}}$. On the other hand, the higher the value of available bandwidth, the greater the path quality tends to be. We conclude, therefore, that the greater the value of $\varphi_{p}$, the better it tends to be the path, considering all the criteria simultaneously.

(1) $$\varphi_{p}=\mu_{1}\bar{awb_{p}}+\frac{1}{\mu_{2}\bar{delay_{p}}}+\frac{1}{\mu_{3}\bar{jitter_{p}}}$$

The Algorithm 2 implements the procedures. The input of the algorithm is the internal state and the IoMT multiuser mobile session identifier created or adapted. As an output, the algorithm defines the IoMT multiuser mobile session transport trees including the paths selected via the Equation (1).

**Algorithm 2:** Definition of the IoMT multiuser mobile session transport trees.

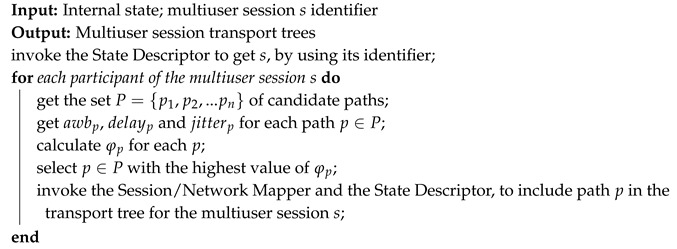



##### Session/Network Mapper

The Session/Network Mapper module applies a mapping function that considers (i) the SDN nodes that connect the content provider to the egress SDN node, for each participant in the IoMT multiuser mobile session; and (ii) branching nodes in the IoMT multiuser mobile session transport trees. This function relies on the principle of group communication to define a set of one-to-many flow rules (for branching nodes) and one-to-one for other nodes (i.e., non-branching) of the softwarized network. A branching node represents the one closest to the edge of the softwarized network and connects more than one participant to the IoMT multiuser mobile session.

The procedures of the module are implemented by the Algorithm 3. The input of the algorithm includes the internal state and the IoMT multiuser mobile session identifier (created or adapted). The algorithm returns the flow rules that will effectively define a segment (i.e., associated with an IoMT multiuser mobile session) of the *QoS-oriented multiuser network*.

**Algorithm 3:** Mapping IoMT multiuser mobile sessions to the multiuser network. **Input**: Internal state; IoMT multiuser mobile session *s* identifier **Output**: Flow rules that define the QoS-oriented multiuser network invoke the State Descriptor to get *s*, by using its identifier; get the IoMT multiuser mobile session transport tree associated with *s*; define the branching node(s); define the one-to-many flow rules (for branching nodes); define the one-to-one flow rules (for non-branching nodes); invoke the State Descriptor to update the description of the IoMT multiuser mobile session *s*; notify the SDN Controller to install the flow rules in each node;

The one-to-many flow rules are defined based on group communication, from the substrate offered by the SDN Controller (i.e., standardized OpenFlow OFPT_GROUP_MOD messages). In these one-to-many rules, each replicated packet will include the host’s IP address of the IoMT multiuser mobile session participant and the corresponding port at the transport level. The other nodes (i.e., non-branching) will receive one-to-one flow rules defined by the module, taking into account message specifications (i.e., OpenFlow OFPT_FLOW_MOD).

After the installation of the flow rules, the content request message, sent by CMIA, will finally be transported, from the ingress node to the content provider on the cloud IoT platform. Based on the request, the content provider then performs its specific operations to serve the CMIA. Finally, the provider initiates the communication session, and the data stream will be managed in the QoS-oriented multiuser network provided by ReMUSiC-Cloud.

### 3.2. ReMUSic-Fog Components and Modules

**Figure 8:**
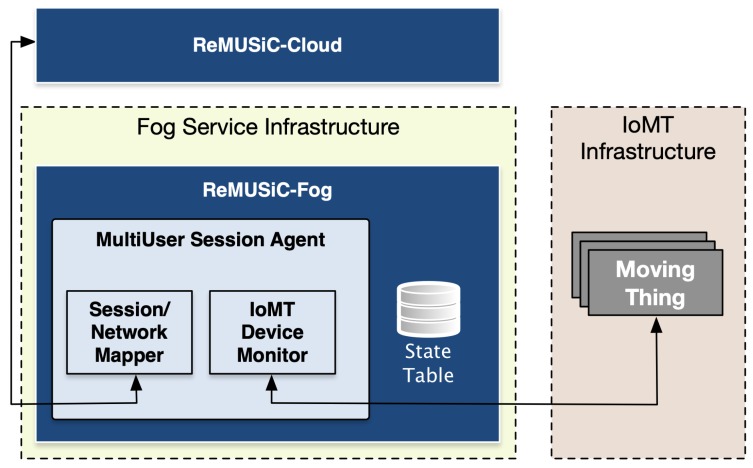
The MultiUser Session Agent (MUSA) component.

In the ReMUSiC framework design, the ReMUSiC-Fog system follows the premise to pursuit a lightweight approach, in a sense to enhance the agility of the entire system and improve the cloud scalability, whereas the ReMUSiC-Cloud assumes must of the control plane complex capabilities. It is assumed that a set of content providers’ applications run in fog nodes in the form of virtual structures, as well as the ReMUSiC-Fog component. It is worth highlighting that the orchestration of such virtualized structures is out of the ReMUSiC control plane scope. Figure 8 introduces the MUSA architecture, raising its components and interfaces with other modules.

In the ReMUSiC-enabled ecosystem, the ReMUSiC-Fog system extends the Session/Network Mapper module ability of the ReMUSiC-Cloud’s MUNO component to participate in MultiUser Session Agent (MUSA), with the goal to speed up group-communication state setup on-site (i.e., locally in the fog node). The Session/Network Mapper module participating in the MUSA component is in charge to maintain locally activated the knowledge of IoMT multiuser mobile sessions on every fog node. This knowledge turns MUSA capable to locally set up group forwarding state (through OpenFlow) without the need to trigger the SDN controller accordingly. On finishing the new group forwarding state setup, MUSA notifies the ReMUSiC-Cloud accordingly to keep activated the knowledge of IoMT multiuser mobile sessions up to date. This is a critical contribution to decentralize the task of setting the forwarding state in the whole ReMUSiC domain. In classical SDN, the SDN controller is the only one responsible for this type of configuration, raising scalability penalties and the subject of performance degradation.

Moreover, the architecture of the MUSA component entails the IoMT Device Monitor (IoMT-DM) participating module. The IoMT-DM is in charge to maintain fog node local-premises knowledge, including: (i) the set of already attached and new IoMT devices; and (ii) the activated IoMT multiuser mobile sessions along with respective IoMT device participants. The IoMT-DM module is also responsible to notify the ReMUSiC-Cloud about the attachment of new IoMT devices, to support quality-oriented resilience procedure accordingly. This resilience assistance (IoMT mobility) stands as a key capability of the ReMUSiC-Fog system, and potentially assists the ReMUSiC-Cloud in the attempt to keep respective connectivity over time. The MUSM component in turn assumes the task of keeping the ReMUSiC-Cloud IoMT multiuser mobile session knowledge always up to date, meaning that an announcement event is invoked whenever either a new IoMT multiuser mobile session is activated or a new IoMT member is added.

## 4. Results

This section presents the evaluation of the operation of the ReMUSiC framework, considering a scenario of CMIAs based on video provided by an IoT platform. The main objective of this evaluation is to analyze the resilience, suitability, feasibility, effectiveness and performance impact through the use of the ReMUSiC framework, in comparison to relevant related work, in maintaining the performance of the IoMT system as a whole.

### 4.1. Evaluation Model

**Figure 9:**
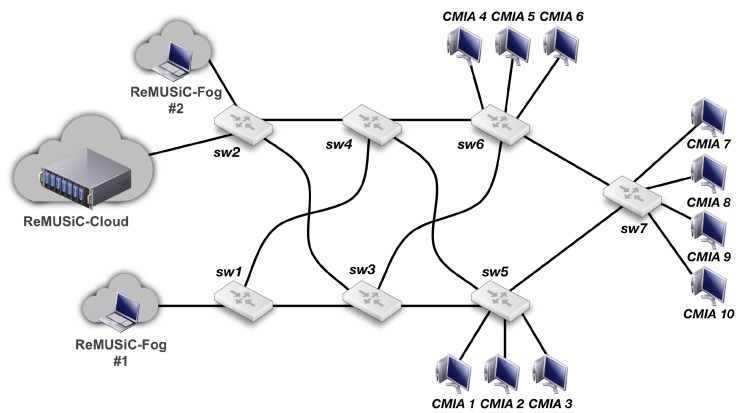
Testbed topology corresponding to the evaluation model.

The evaluations consider a real testbed existing in the Ubiquitous and Pervasive Systems Lab (UPLab), located in the Nucleus of Research and Innovation in Technology (NPITI) of the Federal University of Rio Grande do Norte (UFRN), where the ReMUSiC framework was prototyped to cater precise analysis of its approach. Figure 9 illustrates the testbed configuration, which corresponds to a typical SDN network topology for content provider environments in IoMT [50].

As shown in Figure 9, the network core deploys an open approach through 7 OpenFlow-enabled (V1.3) common-off-the-shelf (COTS) hardware switch nodes, identified as $sw_{1}$ up to $sw_{7}$. These nodes are of type Routerboard Mikrotik RB 951G-2HND set to switching mode interconnected through a meshed-topology of IEEE 802.3 links with 10Mbps of total bandwidth. A total of 10 CMIAs (identified as CMIA 1 up to 10) are in the form of clients running on the hosts directly connected to the edge nodes $sw_{5}$, $sw_{6}$ and $sw_{7}$. These clients are subjected to receive video streaming flows sent from the Video Server (liked to $sw_{1}$), which leverages the IoT FIWARE platform.

All components and modules of the ReMUSiC framework have been implemented by the Northbound API provided by the Floodlight Controller architecture. This allows the ReMUSiC framework to invoke the topology management and gathering of both network statistics and services provided by the SDN Controller features. Such services provide statistics collected from the IoMT Data Plane, which afford the procedures and algorithms of the ReMUSiC-Cloud and ReMUSiC-Fog components.

Following the guidance of relevant literature, two experimental environments are adopted, namely ReMUSiC and SDN Multicast. The set of ReMUSiC experiments, as the name itself suggests, contains the ReMUSiC framework implemented with full fidelity to its definitions. The set of SDN Multicast experiments is based on the approaches defined in [30], which stands out over the other related works by extending the SDN Controller with methods that are based on the Multi-Tree Routing and State Assignment Algorithm (MTRSA) to construct IP multicast trees which, like ReMUSiC, is supposed to optimize total bandwidth consumption in the SDN.

The streaming distribution by the Video Server is controlled by the Evalvid [51] tool, which is capable of rebuilding a received video even when frames are lost during transmission on the network. Based on the recent literature on video quality evaluation [52], a video server application running in the ReMUSiC-Fog nodes #1 and #2 (Video Server) generates streaming with the following properties: MPEG-4 codec, file size 8.09 MB, high resolution of 1920 × 1080, frame rate 24 fps (frames per second), and bitrate (high) of 1 Mbps. The total experiment time is 60 s, and each of the 10 video requests (sent by CMIAs) occurs at a random time between 0 and 10 s of the experiment. These experimental parameters (namely duration of the experiment, characteristics of the video session, number of requests and active video streams) are defined respecting the computational limits associated with Evalvid. Values beyond these limit the evaluation model by issues of limitation of the adopted tools and of the testbed itself. The Real-Time Protocol (RTP) protocol over UDP is used for video streaming.

Aiming to identify the behaviour of the ReMUSiC and SDN Multicast experiment sets when (i) the number of CMIA increases with time; and (ii) a set of IoMT mobility events occur during the experiment time, the following configurations are induced over the experiment time:

- a total traffic load of 100% of the link capacity is induced (denoting a hostile network environment) in the vicinity of the Video Server. Between instant times 0 and 25 s, a 9 Mbps traffic overhead is induced by the Video Server application running at the ReMUSiC-Fog #1. Between experiment times 25 and 50 s, this 9 Mbps traffic overhead occurs only on the link $\left\{sw_{1},sw_{4}\right\}$. This load switching is intended to identify how the solutions adapt to changing the QoS standards (i.e., latency, jitter and bandwidth) in the network. When the offered traffic overhead exceeds the maximum limit of the link capacity, there is no significant change in the pattern of results, which justifies the evaluation model;
- between instant time 28 and 32 s (i.e., close to half the total time of the experiment), the video server application detaches from ReMUSiC-Fog #1 (finishes) and attaches to ReMUSiC-Fog #2, denoting IoMT device mobility event. The video server at ReMUSiC-Fog #2 starts with the first frame immediately after the last frame streamed by ReMUSiC-Fog #1, for continuity). Thus, how ReMUSiC impacts in terms of QoS/QoE on carrying out a resilience procedure can be assessed; and
- after 50 s (i.e., when there is no more traffic overhead), it is sought to evaluate the behaviour of the solutions in a network with low traffic load (i.e., with 10% of link capacity).

The Full Reference (FR) video evaluation model is used, since full access to the original video is required to rebuild the received video. This means that two input files are required for the FR model: the original file (transmitted by the Video Server) and the transmitted file (received by the client). These files are processed by the Video Quality Measurement Tool (VQMT), a software tool capable of evaluating the received video according to different QoE metrics. Table 3 summarizes the main parameters of the experiment in this evaluation model.

The methodology applied for the prototyping assessments entails 10 rounds of tests per each set of experiments (i.e., 10 for ReMUSiC and 10 for SDN Multicast). Results obtained at a confidence level of 95% are traced for analysis, whereby graphs sketch average rates of a respective round of tests.

### 4.2. Metrics

The analysis of session activation considers two related metrics, as described below:

- **Session Setup Time**—in this work, the setup time of a communication session (i.e., IoMT multiuser mobile session, with ReMUSiC, and IP Multicast session, with SDN Multicast) corresponds to the elapsed time (in milliseconds) between the sending of the content request message until the receipt of the first content data packet, for each client; and
- **Control Overhead**—the increase in the amount of signalling messages (e.g., OpenFlow and Internet Group Management Protocol (IGMP) protocols used by the SDN Multicast) tends to impact the session setup time. In this work, the control overhead represents the sum of the number of packets of the OpenFlow and IGMP protocols, hence being directly associated with the scalability of the system.

From QoS, the metrics used to evaluate the solutions follow the recommendations in [53], for the reasons described below:

- **Latency (or End-to-End Delay)**—video streaming has substantial latency requirements. When video packets are delivered to the client in addition to a few hundred milliseconds, it is considered useless for the playback process (play-out) [54];
- **Jitter**—in the case of video streaming (which imposes a heavy load of data traffic on the network), the increase in jitter typically results from network congestion IP, among other reasons [55]. Jitter is a relevant and undesirable factor since it tends to degrade the processing performance (i.e., CPU) of the nodes and increase the packet loss in the network. Knowing that different packets of the same data stream can cross different network paths, it is critical to measure network QoS in terms of jitter; and
- **Throughput**—throughput can be reduced when there is an increase in the number of data streams sharing network links, which can lead to network maximum video playback limits, and thus affect user satisfaction.

The evaluation model considers two QoE metrics widely used by the scientific community to evaluate the level of quality of the video perceived by the user. These metrics were used based on the Recommendation [56], since the CMIAs are highly sensitive in terms of QoS in the network transport, evidencing the strong correlation between QoS and QoE. The metrics considered in the evaluation are described as follows:

- **Structural Similarity Metric (SSIM)**—it measures the similarity between the original video and the video being played back. From the similarity analysis, the SSIM value is measured in the range of [0..1]. When it is 1, the video played on the client side is exactly the same content transmitted by the server, and when it is 0 it represents no correlation [57]; and
- **Video Quality Metric (VQM)**—it is a metric that considers video frame distortion (when comparing brightness, contrast, pixels, and other properties) to check the quality of the video received. The best possible value of VQM is 0 (maximum user satisfaction), when the original and received videos reach the best combination for the human eye, and in real situations, it can reach the value 12 [58].

### 4.3. Session Setup Evaluations

This section presents an analysis on the session setup time by ReMUSiC and SDN Multicast during the experiment time, as more CMIAs request the video.

#### 4.3.1. Analysis of the Session Setup Time

**Figure 10:**
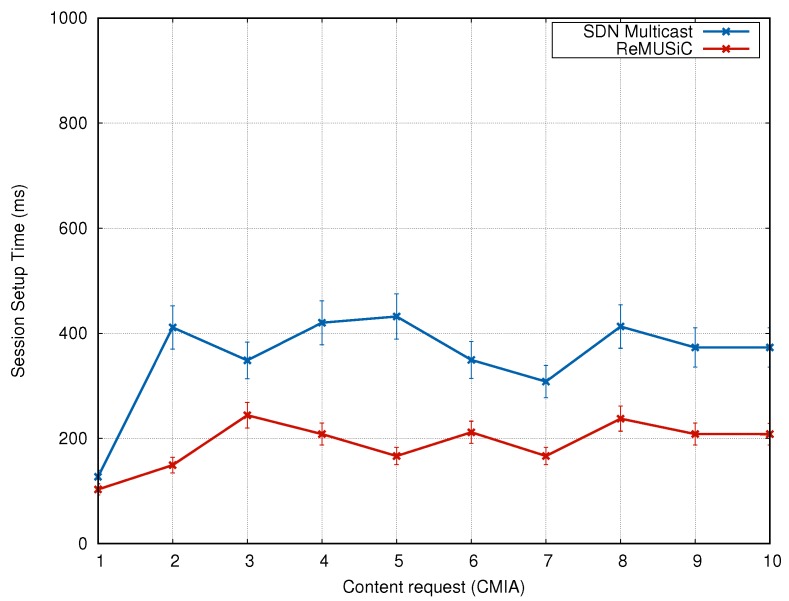
Session Setup Time.

The session setup times achieved by ReMUSiC and SDN Multicast are shown in Figure 10. The *x*-axis shows each content request (which occurs at a random time between 0 and 10 s of the experiment) performed by each of the 10 CMIAs. In the *y*-axis, the session setup time (in milliseconds) for each client is shown.

It can be noted that ReMUSiC has optimized session setup time, in comparison to SDN Multicast. At the beginning of the experiment (i.e., when the first request is handled), the session setup time of both solutions was close (about 160 ms). However, ReMUSiC was able to reduce session setup time for subsequent requests. On average, ReMUSiC gains reached 46% by enabling sessions in an average time of 190 ms, while SDN Multicast enabled sessions at an average time of 355 ms. These gains were mainly due to ReMUSiC’s low control overhead, which allowed for shorter times for receipt/processing (of requests sent by clients) and the effective start of data flow in the QoS-oriented multiuser network.

#### 4.3.2. Analysis of the Control Overhead

**Figure 11:**
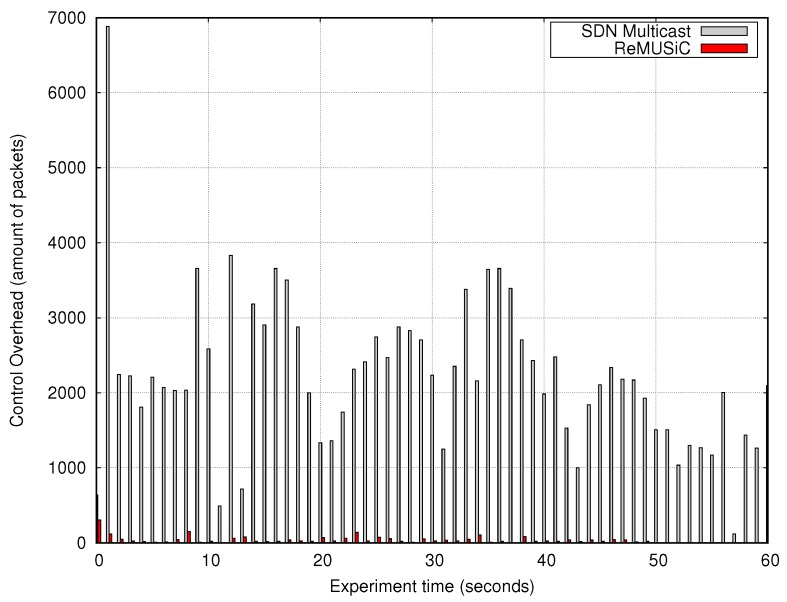
Control overhead.

It can be observed in Figure 11 that ReMUSiC allows for a drastic reduction of the control overhead compared to the set of SDN Multicast experiments, regardless of the instant of time. This huge advantage of the ReMUSiC can be verified in the average value of the control overhead. While ReMUSiC maintained an average of 54 control packets (OpenFlow) per second, the SDN Multicast injected an average of 1910 control packets (OpenFlow and IGMP) per second for its network control operations. Thus, ReMUSiC allows the control overhead to be reduced by about 97.17% in comparison to SDN Multicast. While ReMUSiC only uses OpenFlow messages (via the secure channel, out-of-band, between SDN Controller and OpenFlow nodes) to construct the QoS-oriented multiuser network, the SDN Multicast still uses IGMP (in-band) for the creation and maintenance of the IP multicast trees, which significantly increases the control overhead.

These results suggest that ReMUSiC allows optimizing the consumption of network resources (e.g., the bandwidth of the links, time of processing/storage of nodes) at the same time it handles multiple CMIAs simultaneously. This demonstrates that ReMUSiC ensures greater scalability in IoMT than SDN Multicast. It is also possible to infer that the resource consumption in the Video Server is also optimized with ReMUSiC since it only needs to handle the first content request. All other requests are handled by ReMUSiC components, with total transparency about the cloud IoT platform.

### 4.4. Analysis of QoS

This section discusses the results obtained by the solutions from the QoS at the network level.

#### 4.4.1. Analysis of the Latency

**Figure 12:**
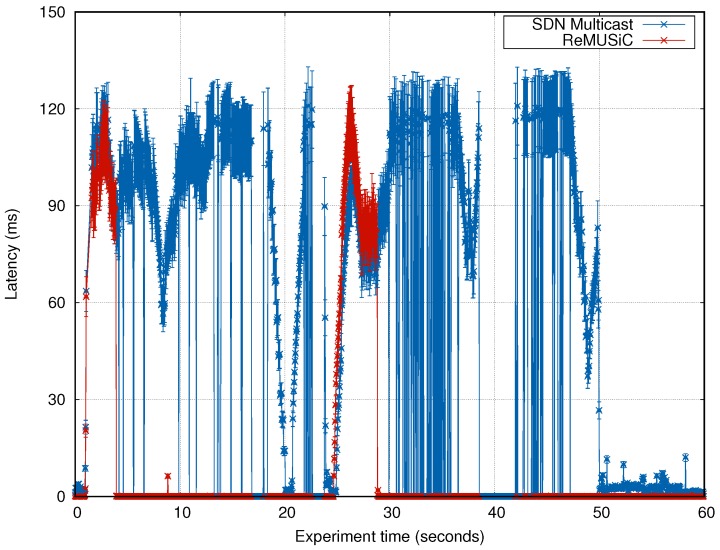
Latency impact in both ReMUSiC and SDN Multicast experiments.

The latencies in the content transport obtained by ReMUSiC and SDN Multicast are shown in Figure 12. ReMUSiC significantly improves the performance impact in terms of latency for most of the time. The averaging rates for the latency that ReMUSiC experiment allows are of 9.20 ms, whereas the SDN Multicast averages 53.66 ms.

The resulting latency impact optimization of ReMUSiC in comparison with the SDN Multicast is of 83%. These gains are a direct consequence of the quality-oriented IoMT multiuser mobile sessions capability, through which CMIAs are grouped into IoMT multiuser mobile sessions and QoS-oriented multiuser network are enforced along best-connected paths over time. ReMUSiC reaches a transient state of operation between instants 0 s and 5 s of the experiment, which occurs due to the update of the IoMT multiuser mobile sessions table. On the basis of this event, ReMUSiC needs to update the multiuser tree, define the flow rules, and enforce the setup of such flow rules on nodes accordingly. SDN Multicast also increases its latency to build the multicast trees in the network, as a function of the IGMP join operations by the CMIAs.

A fluctuation in the latency of both solutions is perceived around 28 s. This fluctuation results from the induced fault in a network link. Because of its resiliency procedure, ReMUSiC detect the degradation of QoS parameters at the network level in paths, recalculates the value of φ from other candidate paths (with better QoS values), and select the best available path to attend IoMT multiuser mobile session participants. Differently, the SDN Multicast continues to forward the data packets along the shortest path (following the Shortest Path First (SPF) algorithm), leading to increased latency.

#### 4.4.2. Analysis of the Jitter

**Figure 13:**
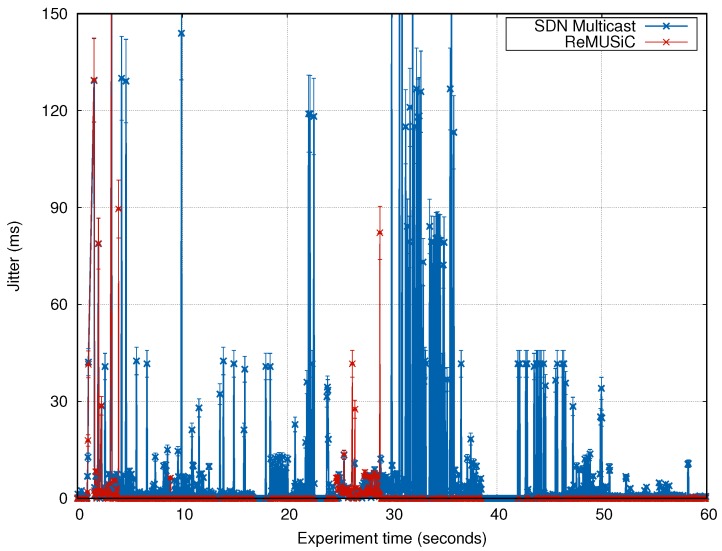
Jitter impact in both ReMUSiC and SDN Multicast experiments.

Figure 13 shows the behaviour of the Jitter during the experiment time, where ReMUSiC achieves superior performance over SDN Multicast. The average value of the Jitter of the ReMUSiC was 0.38 ms, while the average value obtained by the SDN Multicast was of 4.02 ms, evidencing that the average gain that ReMUSiC obtains is of 90%.

It is even noted that ReMUSiC (other than SDN Multicast) maintains the Jitter below 30 ms (a requirement for multiple video-based CMIA) most of the time. The reason for all this gain from ReMUSiC is due to the ability to redirect packets to less congested paths. In addition, lower control overhead contributes to better link bandwidth utilization in ReMUSiC.

#### 4.4.3. Analysis of the Throughput

**Figure 14:**
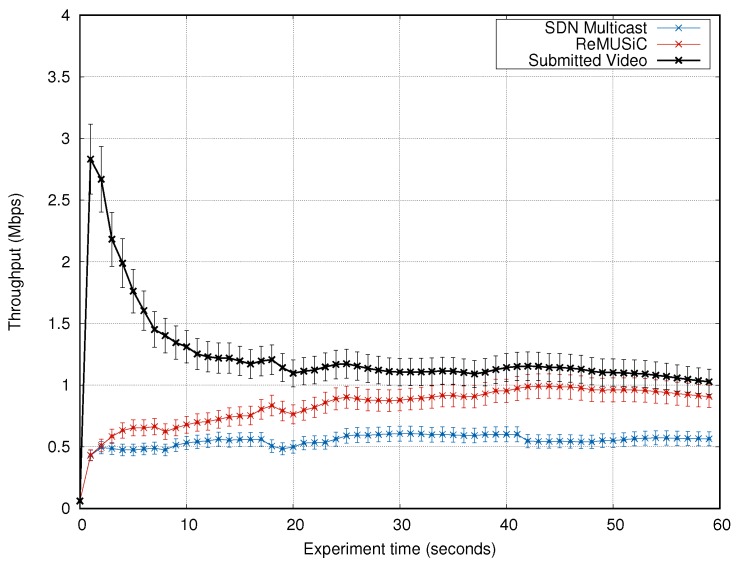
Throughput impact in both ReMUSiC and SDN Multicast experiments.

The results of the throughput are shown in Figure 14. The throughput that ReMUSiC obtains is closer to the transmission rate of the original video, in comparison to SDN Multicast. After the transient state in the network (i.e., at the beginning of the experiment), the throughput of ReMUSiC increases gradually throughout the experiment time, reaching values closer to 1 Mbps by 20 s. Meanwhile, the flow of the SDN Multicast stays a little over 500 Kbps most of the time.

In the average during the experiment, ReMUSiC improves data throughput by about 30% over the SDN Multicast (0.83 Mbps and 0.54 Mbps respectively). These ReMUSiC gains in data throughput impact are justified by a set of factors. On one hand, there is a direct correlation between control overhead and throughput. With ReMUSiC, the control overhead is out-of-band (unlike SDN Multicast, which uses IGMP in-band messages). On the other hand, ReMUSiC relies on the routing metric that selects the best paths in terms of QoS, allowing to use those paths that offer less latency/jitter.

### 4.5. Analysis of QoE

In this section, we discuss the results obtained by the solutions from the QoE, since it provides an accurate view of the quality of video transmission from the user perspective [59].

#### 4.5.1. Analysis of the SSIM

**Figure 15:**
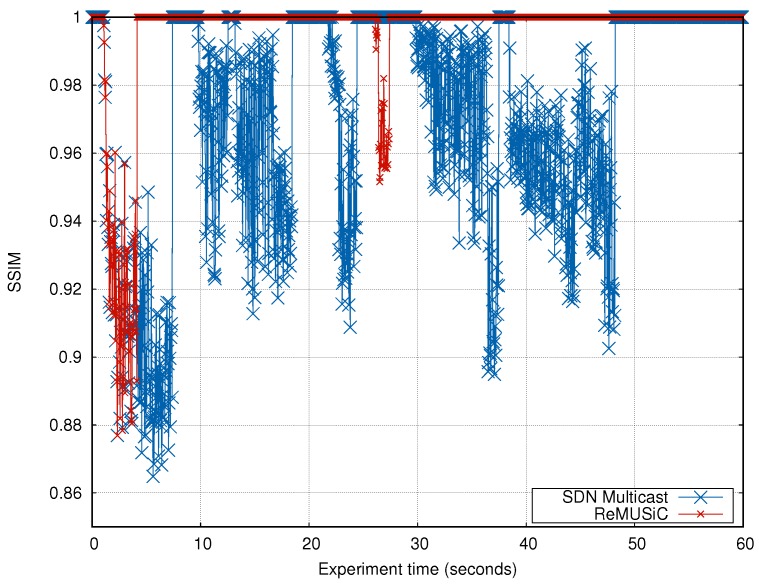
SSIM impact in both ReMUSiC and SDN Multicast experiments.

Figure 15 shows the impact behaviour of the SSIM of ReMUSiC and SDN Multicast. It reveals that ReMUSiC outperforms SDN Multicast, whereby the former maintains a very close correlation between the original video and the files received by the participants of the IoMT multiuser mobile session during most of the experiment time.

It should be noted that the traffic load on the network reaches 100% of the link capacity, between instants 0 s and 50 s. While SDN Multicast achieves an SSIM of 0.92, on average, ReMUSiC obtains an average rate of 0.99. These SSIM improvements (7% on average) result from ReMUSiC’s quality-oriented IoMT multiuser mobile session management, and the selection of better-connected paths in the QoS-oriented multiuser network, even in front of a network saturated with hostile load.

Figure 15 also sketches that ReMUSiC achieves the best possible correction (i.e., SSIM equals 1), except at two specific moments: (i) during the transient period of the network (at the beginning of the experiment), and (ii) at the time of the induced fault (between instants 28 s and 32 s). In those specific periods, where ReMUSiC performs the reconfiguration of the IoMT multiuser mobile session (and the reconfiguration of paths) due to reduced network performance, a portion of the data packets was lost, thus affecting the SSIM value. However, these results show that ReMUSiC provides a resilient content transport service to maintain the quality of IoMT multiuser mobile sessions, even after a networking event. On the other hand, the SDN Multicast configuration remained using the smallest path (with lower quality) for most of the time, leading to more significant losses in terms of SSIM. From moment 50 s, where the traffic load on the network is low (10% of link capacity), both solutions achieve the best SSIM values, as expected.

#### 4.5.2. Analysis of the VQM

**Figure 16:**
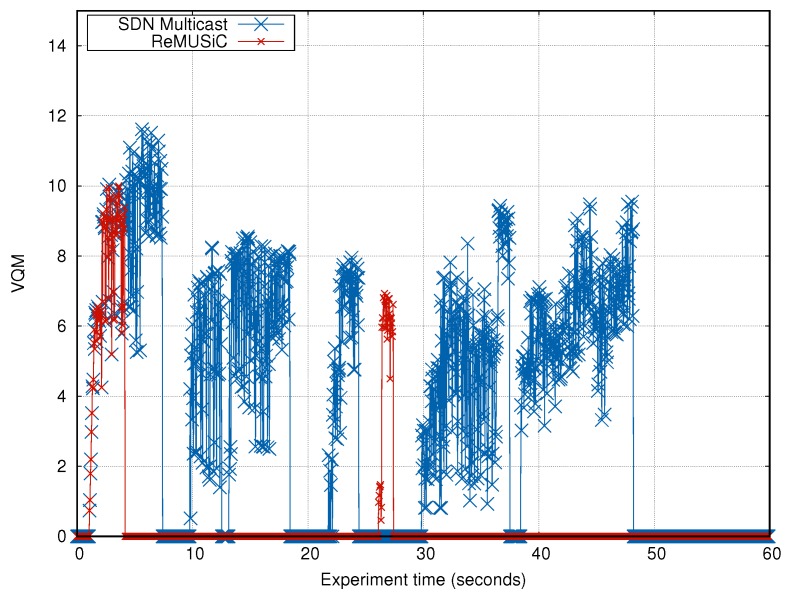
VQM impact in both ReMUSiC and SDN Multicast experiments.

In Figure 16, the VQM outcomes that both solutions raise over the experiment time are shown. It can be seen that the VQM obtained by ReMUSiC is better than that obtained by the SDN Multicast for most of the time. The average value of VQM reached by the SDN Multicast is 0.47. Otherwise, ReMUSiC’s VQM is much better, reaching the average value of 3.55.

This represents ReMUSiC gains around 86% over the SDN Multicast result. Besides, it may be noted that ReMUSiC offers the best possible quality (i.e., VQM equals 0) out of the transient state and after the network fault, hence highlighting the benefits of its resilience procedures. Knowing that VQM offers a higher correlation with QoE than SSIM [60], these results strongly indicate better QoE with ReMUSiC, compared to SDN Multicast.

## 5. Conclusions

This paper proposes a holistic framework, called Resilient MultiUser Session Control (ReMUSiC), which deploys emerging softwarization and cloudification technologies to afford flexible, optimized and self-organized control plane perspectives. ReMUSiC innovates on offering a quality-oriented resilience mechanism to respond to network dynamics events (i.e., failure and mobility) by (re)adapting IoMT multiuser mobile sessions. Moreover, ReMUSiC adopts a softwarized networking control plane to fetch current network state and set up resources in runtime to keep IoMT multiuser mobile sessions always best-connected and best served. A cloudification approach allows a robust environment, through which cloud- and fog-systems internetwork to cater to performance-enhanced capabilities.

The experiments performed on a real testbed that implements both the ReMUSiC framework and the signalling architecture of relevant related work, called SDN Multicast, demonstrate its significant effectiveness in connecting multiuser sessions sensitive to QoS in the presence of network events. The results show that ReMUSiC, in comparison to SDN Multicast, allows: (i) reducing control signalling overhead by about 97%; (ii) optimizing the average session setup time of CMIA multiuser sessions by 46%; (iii) optimizing 83% in terms of latency, and 90% of jitter, during content transport; (iv) improving the performance of data flow by about 30%; (v) and substantially outperforms QoE to CMIA sessions, revealing a performance rate of 7% for SSIM and 86% for VQM. Therefore, the results demonstrate the benefits that the use of the ReMUSiC framework brings to cases of CMIA use in IoMT systems.

Future works include defining component to allow adaptation of video properties, such as codec and bitrate, consumed by a CMIA over its lifecycle, considering the resources at the session and network levels in the QoS-oriented multiuser network. Moreover, we aim at experiencing the ReMUSiC framework in a Smart City environment, to evaluate it in a real critical scenario and with a direct social impact.

## Figures and Tables

**Table 1:** Analysis of existing solutions.

Solution	Session-Level Semantics	Multiuser Communication	Quality-Oriented Control Plan	Resilience
Nam et al. [26]	–	–	–	–
Llopis et al. [18]	–	–	–	–
Chen et al. [19]	–	–	X	–
Jang et al. [27]	–	–	–	–
Rafael and Varese [28]	–	–	–	–
Multiflow [29]	–	Multicast IP	–	–
MTRSA [30]	–	Multicast IP	–	–
Tang et al. [32]	–	Multicast IP	–	–
Huang et al. [20]	–	Multicast IP	–	–
Noghani and Sunay [31]	–	Multicast IP	–	X
OpenE2EQoS [21]	–	Multicast IP	–	X
Soni et al. [22]	–	Multicast IP	–	X
SDM^2 Cast [24]	–	Group/SDN	–	–
Hungyo and Pandey [25]	–	Group/SDN	–	–
ReMUSiC	X	Group/SDN	X	X

**Table 2:** Example of semantic knowledge assigned to multiuser sessions.

Identifier	Producer	Content	Participants
1	$id_{fog_{1}}$	$video_{1}$	$\left\{cmia_{1},cmia_{2}\right\}$
2	$id_{fog_{2}}$	$video_{2}$	$\left\{cmia_{3},cmia_{4}\;cmia_{5}\right\}$
3	$id_{fog_{3}}$	$video_{3}$	$\left\{cmia_{6},cmia_{7}\right\}$

**Table 3:** Parameters of the experiment.

Parameter	Value
Amount of CMIA	10 CMIAs
Link capacity	10 Mbps
Traffic load	Up to 100% of the link capacity
Video codec	MPEG-4
Video size file	8.09 MB
Video resolution	1920 × 1080
Frame rate	24 fps
Bitrate	1 Mbps
Transmission protocols	RTP/UDP
Evaluation model	Full Reference
Experiment time	60 s
